# Periodontal health of endodontically treated molars restored with composite CAD/CAM endocrowns versus stainless steel crowns in Egyptian children: a randomized controlled trial

**DOI:** 10.1186/s12903-026-08077-0

**Published:** 2026-04-18

**Authors:** Basheer Ali Mabkhot, Sheriene Ezz Eldin Taha, Mostafa Hussein Kamel, Shaimaa Mohamed Sabry

**Affiliations:** 1 Faculty of Dentistry, Ibn Al-Nafis University, Sana’a, Yemen; 2 The First Pediatric Dental Clinic, Sana’a, Yemen; 3 Pediatric Dentistry and Dental Public Health, Faculty of Dentistry, Cairo University, Giza, Egypt; 4 Misr International University, Cairo, Egypt

**Keywords:** CAD/CAM, Endocrown, Egypt, Pediatric dentistry, Periodontal health, Stainless steel crown

## Abstract

**Background:**

Subgingival crown margins have been linked to chronic gingival inflammation in pediatric patients. This clinical trial evaluated periodontal outcomes in first permanent molars that had undergone root canal therapy and were subsequently restored with either CAD/CAM Composite Endocrowns or stainless steel crowns (SSC).

**Patients and methods:**

Twenty-four children aged 10–13 years diagnosed with extensive caries in their first permanent molars were recruited from the Pediatric Dentistry clinics at the Faculty of Dentistry, Cairo University. Participants were randomly allocated to one of two treatment arms, where they received either a CAD/CAM Composite Endocrown or an SSC. The primary outcome was periodontal health assessed by Probing Pocket Depth (PPD). Secondary outcomes included Gingival Index (GI), Plaque Index (PI), and radiographic healing evaluated using the Periapical Index (PAI). Clinical parameters were recorded at baseline and at 1 week, 3, 6, 9, and 12 months. Statistical analysis was performed using appropriate parametric and non-parametric tests with a significance level set at *p* ≤ 0.05.

**Results:**

At baseline, both groups were comparable in age, gender, and all clinical indices (*p* > 0.05). During follow-up, mean PPD values were significantly lower in the Endocrown Group than in the SSC Group at all time points (*p* < 0.001). GI values were significantly lower for Endocrowns at 1 week and 6 months (*p* = 0.031 and 0.004, respectively). PI values showed no significant intergroup difference (*p* > 0.05), though both groups improved over time. Radiographically, both groups demonstrated favorable periapical healing with no significant differences in PAI scores after 12 months. Inter- and intra-observer reliability was excellent (ICC > 0.8).

**Conclusion:**

Within the 12-month follow-up period, composite CAD/CAM Endocrowns were associated with significantly reduced probing pocket depth and healthier gingival conditions compared with SSCs, which suggests that CAD/CAM Endocrowns offer a biologically conservative interim restoration for molars treated endodontically in pediatric patients.

**Clinical trial registration:**

ClinicalTrials.gov, NCT05250609. Registered 22 February 2022. Retrospective registration. https://clinicaltrials.gov/study/NCT05250609.

**Supplementary Information:**

The online version contains supplementary material available at 10.1186/s12903-026-08077-0.

## Introduction

In the transition from primary to permanent dentition, the first permanent molars (FPMs) are typically the earliest to emerge. During this mixed dentition stage, oral hygiene tends to be suboptimal due to a combination of factors, including the child’s carefree age, emotional stresses, high-frequency consumption of refined carbohydrates and sticky food substances, alongside the natural transition from primary to permanent dentition. Consequently, there is a higher likelihood of the FPMs being affected [1].

In pediatric populations, the most frequently observed gingival disorder is chronic marginal gingivitis, typically attributed to insufficient oral hygiene or dental procedures resulting from poorly fitting crowns and from crown placement subgingivally, which allows plaque to accumulate in the region [2]. A distinct correlation exists between periodontal health and the adherence of patients to self-administered plaque control and periodontal maintenance following prosthodontic treatment [3]. A clinical study has reported that, when fixed single crowns are fabricated with appropriate marginal adaptation and patients maintain satisfactory oral hygiene, the periodontal condition of crowned teeth is generally comparable to that of the contralateral untreated natural teeth [4].

Stainless steel crowns (SSCs) are cost-effective, durable, and can fully cover the tooth. Their placement is relatively simple and requires minimal technical precision. Therefore, the use of SSCs is recommended as a temporary restorative option for extensively compromised FPMs in pediatric patients [5–9]. However, it is important to consider that the disadvantage of using SSCs is their limited aesthetic appeal, especially for patients who prioritize their appearance, and a potential negative impact on periodontal tissues [8, 10].

Stainless steel crowns require circumferential tooth preparation, including occlusal reduction and proximal slicing to allow proper seating of the preformed crown [9]. Their margins are commonly placed slightly subgingivally to achieve retention and full coverage [9]. Biomechanically, SSCs function as full-coverage restorations that rely mainly on mechanical retention and cementation rather than adhesive bonding [6]. Although they provide durability and structural protection, their margin location and contour may influence plaque accumulation and periodontal response in pediatric patients [10].

Numerous studies have evaluated the periodontal condition of teeth with SSCs. Geduk et al. (2023) assessed the Plaque Index (PI) and Gingival Index (GI) in FPMs restored with either preformed zirconia crowns (PZCs) or SSCs, finding that SSCs were associated with elevated PI and GI scores compared to PZCs [10]. Also, a study suggests that SSCs have minimal periodontal complications in the medium-term [8]. However, a study concluded that, in children exhibiting proper oral hygiene practices, the use of stainless steel crowns for FPMs does not compromise gingival health [5].

A conservative method for restoring occlusal integrity in cases of substantial coronal loss—up to half of the structure—is the use of endocrown restorations [11]. In cases characterized by extensive coronal damage and constrained occlusal clearance, endocrowns are considered an appropriate restorative option [11]. The design concept of endocrowns is to prioritize preserving healthy tooth structures by utilizing the cavity itself for a strong restoration [11]. Additionally, the cervical margins of the endocrown are designed to remain supragingival whenever possible, improving cleanability and supporting periodontal health [12]. These supragingival margins enhance plaque control and protect the periodontium, while helping prevent violations of the biological width around the restored tooth [12].

Composite computer-aided design/computer-aided manufacturing (CAD/CAM) Endocrowns follow a more conservative preparation philosophy [12]. Retention is achieved through bonding within the pulp chamber, combined with adhesive attachment to enamel and dentin [13]. Preparation emphasizes preservation of cervical tooth structure and a butt-joint margin design, ideally maintained at a supragingival level to facilitate hygiene and protect the biological width [14, 15]. Biomechanically, endocrowns distribute occlusal forces through adhesive integration with the remaining tooth structure, potentially reducing stress concentration and periodontal irritation [16].

While SSCs are widely used in pediatric dentistry, their subgingival margins may contribute to plaque accumulation and periodontal inflammation [5]. In contrast, composite CAD/CAM endocrowns offer a more conservative alternative [11, 12], yet limited clinical evidence exists regarding their periodontal impact in children [17, 18]. Because margin location and restoration contour inherently differ between SSCs and endocrowns, this study was designed as a pragmatic comparison of two clinically used restorative strategies, where these differences may influence periodontal response.

Therefore, the primary aim of this randomized controlled trial was to compare the periodontal condition of endodontically treated first permanent molars restored with Composite CAD/CAM Endocrowns versus SSCs in children, using probing pocket depth (PPD). The secondary aims were to compare additional periodontal indices (gingival index [GI] and plaque index [PI]) and to evaluate periapical status radiographically using the periapical index (PAI) over 12 months.

The null hypotheses of this study were that there would be no statistically significant difference between composite CAD/CAM endocrowns and stainless steel crowns in probing pocket depth (PPD) over the 12-month follow-up period, and that there would be no statistically significant difference between the two restorative approaches in the secondary outcomes, namely plaque index (PI), gingival index (GI), and radiographic periapical status assessed using the periapical index (PAI), throughout the same follow-up period.

## Patients and methods

### Study settings

This investigation was carried out at the Faculty of Dentistry, Cairo University, within the Department of Pediatric Dentistry and Dental Public Health. The research followed a randomized controlled trial framework, using a parallel-group design with an equal 1:1 allocation ratio.

### Ethical considerations

The study received ethical clearance from the Research Ethics Committee of the Faculty of Dentistry at Cairo University (Reference: 4-3-22; approval granted 29 March 2022), and all procedures were conducted in accordance with the approved protocol. Written informed consent was obtained from each child’s parent or legal guardian before enrollment. This trial was retrospectively registered at ClinicalTrials.gov (NCT05250609) on 22 February 2022. Patient recruitment commenced only after ethical approval was granted, and no participants were enrolled prior to ethics clearance.

### Sample size calculation

To calculate the appropriate sample size for the present randomized controlled trial, data from our previously conducted pilot investigation (*n* = 24 restorations; 12 per group) were used as the reference. The primary outcome for sample size estimation was probing pocket depth (PPD) at 12 months. In the Endocrown group, the mean ± standard deviation of PPD after 12 months was 1.73 ± 0.49 mm, while in the SSC group it was 2.86 ± 0.67 mm, corresponding to an effect size (Cohen’s d) of 1.92.

Using a two-sided independent t-test with a significance level (α) of 0.05 and 80% power (β = 0.20), the minimally required sample size was calculated to be 6 participants per group using G*Power software (version 3.1.9.7). To compensate for potential dropouts during follow-up, the sample size was increased, and 12 participants were ultimately allocated to each group, resulting in a total of 24 restorations.

### Study design, recruitment, and grouping

The study was designed as a prospective, randomized, controlled clinical trial, consisting of two parallel groups with an equal 1:1 assignment ratio.

Patients were sourced from the outpatient clinic of the Pediatric Dentistry and Dental Public Health Department at Cairo University. Recruitment was carried out continuously until the required sample size had been achieved. Demographic information, medical, and dental histories were collected from each patient’s parent/guardian and recorded in their diagnostic charts. Prior to the commencement of the trial, written consent was secured from all caregivers, encompassing all ethical considerations related to the trial.

The study enrolled 24 children, who were allocated into two equal groups of 12 through a randomized assignment process. Participants in the intervention arm received composite CAD/CAM endocrowns, whereas those in the control arm were restored with SCC.

### Eligibility criteria

Participants in this trial met specific eligibility requirements. The criteria for inclusion and exclusion are detailed in Table 1.

**Table 1:** Eligibility criteria

Inclusion criteria:	Exclusion criteria:
1. Children aged 10 to 13 years who demonstrated cooperative behavior during dental visits.	1. Excessive mobility.
2. Mature lower FPMs presenting with either necrotic pulp or irreversible pulpitis.	2. Children with underlying systemic disease.
3. Teeth exhibiting at least two to three intact coronal walls.	3. Children with special health care needs.
4. Participation required a documented agreement from a parent or legal guardian	4. Poor oral hygiene
	5. Children with occlusal abnormalities or any parafunctional habits
	6. The presence of both internal and external root resorption.

### Randomization (sequence generation)

The study adhered to the CONSORT (Consolidated Standards of Reporting Trials) guidelines. Randomization was performed after eligibility confirmation and prior to baseline assessment and intervention. A computer-generated random sequence (https://www.randomizer.org/) was created by an independent study coordinator to allocate participants in a 1:1 ratio into two groups (A and B).

**Fig. 1:**
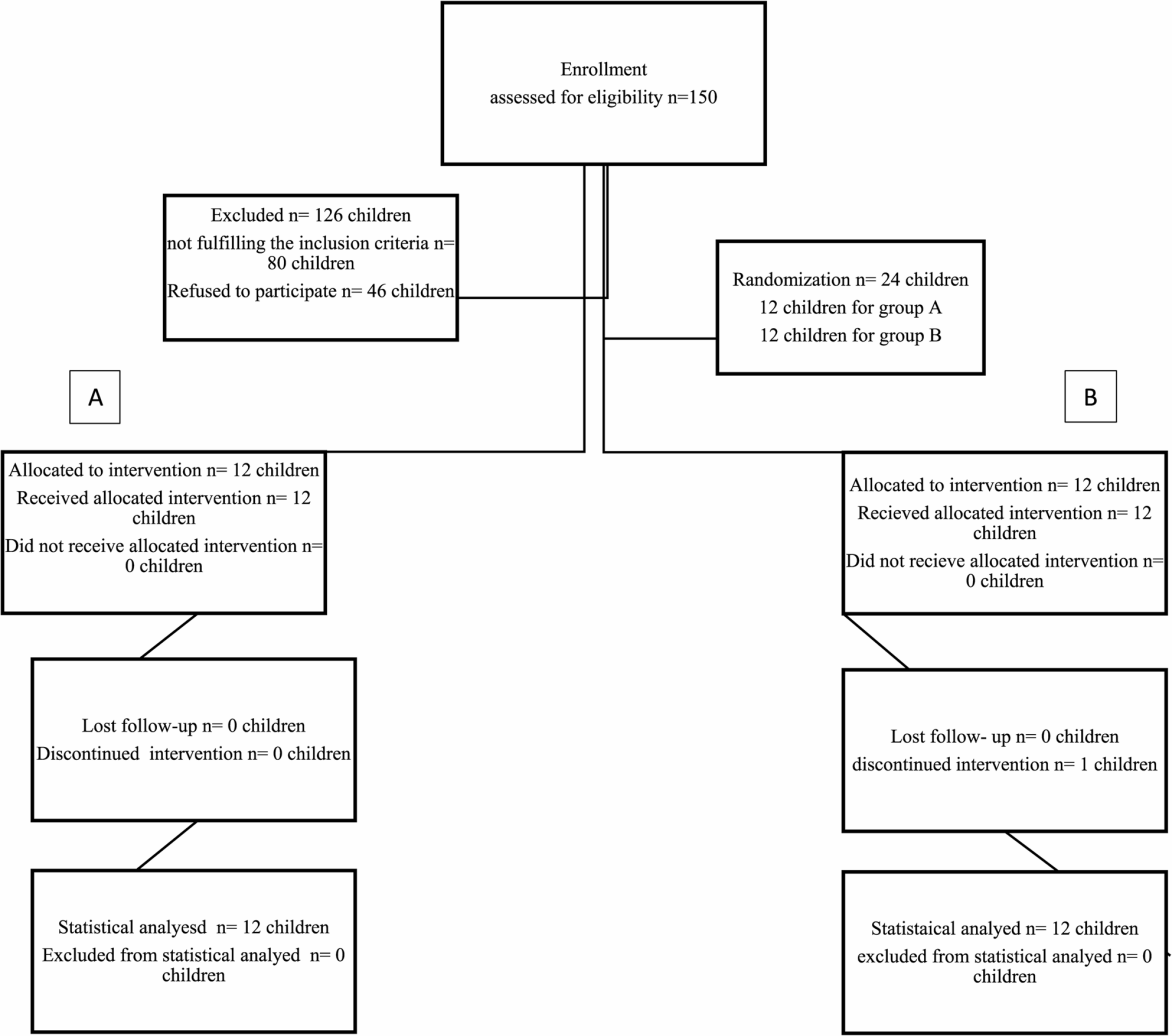
Diagram illustrating participant enrollment, the randomization process, group allocation, follow-up schedule, and data analysis steps in accordance with CONSORT recommendations

Allocation concealment was ensured using sequentially numbered, opaque, sealed envelopes prepared by the coordinator. The envelopes were opened after enrollment to determine the assigned restorative approach. Group A (Endocrown Group) underwent preparation for composite CAD/CAM endocrowns, whereas Group B (SSC Group) received stainless steel crowns (Fig. 1).

### Blinding

To minimize detection bias, the statistician responsible for data analysis was blinded to group allocation throughout the study. Baseline periodontal assessments were conducted by two independent evaluators who were blinded to treatment allocation.

Due to the distinct visual characteristics of stainless steel crowns (metallic appearance) and composite CAD/CAM endocrowns (tooth-colored appearance), blinding of outcome assessors during follow-up evaluations was not feasible. To mitigate potential bias, standardized measurement protocols were strictly followed, and each assessment was performed twice with a five-day interval to verify intra- and inter-observer reliability.

### Clinical steps

A dental unit (Knight; Midmark; Dayton, OH, USA) was utilized during the clinical assessment and management of the patients. Radiographic imaging was performed using a digital X-ray system equipped with a phosphor plate (Scan eXam; KaVo Dental; Biberach, Germany) for both pre- and post-treatment evaluation. Endodontic procedures were completed with the aid of an apex locator (Woodpex III Plus-L-U; Woodpecker; Guilin, China), along with a rotary endodontic motor (E-com Cordless Endo Motor; Woodpecker; Guilin, China) and rotary instrumentation (M-Pro; China).

In the Endocrown Group, treatment was completed over three appointments, whereas the SSC Group required two. For both groups, the first and second appointments were spaced approximately two weeks apart to allow for pain evaluation. In the intervention group, an additional 24–48-hour interval was scheduled between the second and third visits.

#### The first visit for both groups

Root canal therapy (RCT) was conducted in accordance with the 2016 clinical protocols issued by the American Association of Endodontists for the management of irreversible pulpitis and necrotic pulp in permanent molars [19]. Preoperative diagnosis was established through clinical examination, including percussion and cold pulp vitality testing, in addition to radiographic evaluation.

The remaining coronal walls were clinically assessed to confirm the presence of at least two sound walls with adequate cervical thickness to support the planned restoration. Local anesthesia (4% articaine with 1:100,000 epinephrine) was administered, and rubber dam isolation was applied. Access cavity preparation was performed using sterile round and fissure diamond burs. Working length was determined using an apex locator and confirmed with a size 15 K-file.

Canal preparation was carried out using manual K-files (sizes 10–25) in combination with rotary instrumentation (M-Pro system), with irrigation using 2.5% sodium hypochlorite between files, followed by 17% ethylenediaminetetraacetic acid EDTA and saline. Obturation was completed using gutta-percha with a resin-based sealer (ADSEAL; Meta Biomed; Cheongju, Republic of Korea) via lateral condensation. A postoperative radiograph was taken to verify obturation quality, which was considered acceptable when the filling terminated within 0–2 mm of the radiographic apex and exhibited uniform density without visible voids. The access cavity was temporarily sealed (Cavit; 3 M ESPE; St. Paul, MN, USA) until the definitive restorative phase.

#### The second visit for the SSC group

After completing the root canal procedure, a Tofflemire matrix band (Tofflemire; Waterpik; Fort Collins, CO, USA) was applied, and the tooth was restored using glass ionomer cement (Medicem; Munich, Germany). Next, suitable sizes of SSCs (3 M Stainless Steel Crowns for Permanent Molars; 3 M; St. Paul, MN, USA) were selected. The mesiodistal dimension at the proximal contact area was taken with a caliper prior to crown preparation to determine the appropriate crown size.

For crown preparation, approximately 1.5–2 mm of occlusal reduction was carried out, allowing easier access for proximal reduction, followed by mesial and distal surface reduction. The preparation walls were kept conservative, with a slight taper, and the finishing margin was shaped into a smooth feathered edge positioned just below the free gingival margin. All sharp internal line angles were rounded to prevent crown binding during placement. The crowns were then luted using glass ionomer cement (Medicem; Munich, Germany) [20].

#### The second visit for the endocrown group

For cavity preparation, local anesthesia was administered, followed by placement of a rubber dam isolation. The temporary restoration was removed using a fissure diamond bur. The pulp chamber was then cleaned of any remaining sealer using a cotton pellet moistened with alcohol. Subsequently, the chamber was etched with 37% phosphoric acid (Etching Gel; Meta Biomed; Cheongju, Republic of Korea), after which a bonding agent (All-Bond Universal; BISCO; Schaumburg, IL, USA) was applied. The canal orifices were finally sealed with a flowable composite material (Nano Hybrid Flowable Composite Resin; Meta Biomed; Cheongju, Republic of Korea).

For tooth preparation, a tapered stone was first used to establish depth grooves to guide the required occlusal reduction. The occlusal surface was then shaped using a wheel stone to achieve approximately 1.5 mm of clearance, ensuring a flat surface suitable for a butt-joint design. The transition area between the occlusal portion and the mesial or distal cavity was smoothly rounded [17, 21].

The internal cavity walls were flared using a tapered stone with a rounded tip (TF-21/WR-13; AZDENT; China). A retraction cord was then inserted to provide gingival retraction for the impression procedure. The depth of the pulp chamber and the thickness of the prepared tooth walls were assessed with a periodontal probe (Perio Prob; Dentart Instruments; Sialkot, Pakistan) to confirm that the remaining structure measured at least 3 mm [22].

An impression of the prepared tooth was obtained using a condensation silicone material (Zetaplus; Zhermack; Badia Polesine, Italy). An interocclusal record was obtained using a vinyl polysiloxane (VPS) bite registration material (O-Bite; DMG; Hamburg, Germany) to accurately capture the maxillomandibular relationship. After the dental stone was mixed and poured, the resulting models and the bite registration were sent to the laboratory for fabrication of the composite CAD/CAM endocrown.

In the laboratory, the composite block (BreCAM High Impact Polymer Composite; Bredent; Senden, Germany) was utilized to mill the composite CAD/CAM endocrown. The stone models were digitized using the scanner (Medit T710; Medit; Seoul, Republic of Korea) with CAD software (Exocad; Exocad GmbH; Darmstadt, Germany). The resulting scan files were then imported into the dedicated design software for further processing. The finalized restoration was fabricated using a milling machine (DWX-52D; Roland DG; Hamamatsu, Japan). All endocrowns in the study were produced using this equipment [22].

#### The third visit for the endocrown group

For cementation of the composite CAD/CAM endocrown, rubber dam isolation was first established following the administration of local anesthesia. The tooth surface was etched with 37% phosphoric acid for 40 s, rinsed, and gently dried. A universal bonding agent (All-Bond Universal; BISCO; Schaumburg, IL, USA) was then applied according to the manufacturer’s instructions and polymerized for 40 s. The finished endocrown restoration was conditioned and bonded extraorally using the same adhesive protocol. The endocrown was seated using a self-adhesive, dual-cure resin cement (Nova Resin Cement; IMICRYL; Konya, Turkey) following contemporary adhesive cementation recommendations for indirect restorations [13, 14].

After seating, excess resin cement was removed during the gel phase using a microbrush and dental explorer, and dental floss was gently passed through the mesial and distal contact areas to eliminate interproximal remnants. Final polymerization was then completed using a light-emitting diode (LED) light-curing unit (LED.B; Woodpecker; Guilin, China; light intensity approximately 1000 mW/cm²) for 40 s from multiple directions to ensure complete setting of the cement.

The occlusion was evaluated with articulating paper, and any high points were reduced and polished to ensure comfort during biting. To maintain consistency throughout the study, a single operator performed all clinical procedures [22].

### Outcome assessment

Outcome evaluation was performed preoperatively and postoperatively at 1 week, 3, 6, 9, and 12 months.

#### Primary outcome

##### Probing pocket depth (PPD)

Measurements were recorded at the mesiobuccal, distobuccal, mesiolingual, and distolingual sites of each tooth. The periodontal probe (Perio Prob; Dentart Instruments; Sialkot, Pakistan) was carefully inserted into the gingival sulcus, kept parallel to the tooth’s long axis, and moved in a gentle walking motion to obtain accurate depth readings [5].

#### Secondary outcomes

##### Plaque Index (PI)

Although PI was not specified in the initial protocol draft, it was added before participant enrollment and before baseline assessment to control for plaque-related confounding. Therefore, PI was recorded at baseline (pre-operative) and at all follow-up visits.

The Löe and Silness Plaque Index (PI) was applied to assess gingival health by identifying areas where plaque accumulates. Each tooth surface—mesial, distal, buccal, and lingual—was given a score from 0 to 3 based on the amount of plaque present, as outlined below:

- Score 0: No detectable plaque in the gingival area.
- Score 1: A thin layer of plaque located along the free gingival margin and adjacent tooth surface, detectable only upon probing.
- Score 2: A moderate amount of soft deposits within the gingival pocket, on the gingival margin, or on neighboring tooth surfaces, visible without magnification.
- Score 3: A heavy accumulation of soft debris in the gingival pocket and/or on the gingival margin and adjacent tooth surface [23].

##### Gingival Index (GI)

The Löe and Silness Gingival Index was employed to assess the gingival condition by identifying areas exhibiting inflammation. Evaluation was performed on the facial cervical or gingival surfaces of the selected teeth, using a scoring scale from 0 to 3, described as follows:

- Score 0: Gingiva appears healthy with no observable inflammation.
- Score 1: Mild inflammation, reflected by slight color alteration, minimal edema, and absence of bleeding during probing.
- Score 2: Moderate inflammation, characterized by noticeable redness, edema, glazing, and bleeding upon probing.
- Score 3: Severe inflammation, showing marked redness and edema, ulceration, and a tendency for spontaneous bleeding [23].

No professional scaling was performed during the study period unless clinically indicated; participants received standardized oral hygiene instructions at each follow-up visit.

### Radiographic pathological findings

Radiographic evaluations were performed preoperatively and again at 1 week, 6 months, and 12 months after treatment. The condition of the periapical tissues was assessed using the Periapical Index (PAI), which is a 5-level radiographic scoring method used to determine the presence and degree of periapical pathology, as shown in Table 2 [24]. Although PAI scoring was not specified in the initial protocol draft, it was incorporated prior to participant enrollment and baseline assessment to standardize radiographic evaluation during follow-up.

**Table 2:** Description of the Periapical Index (PAI) scoring criteria

PAI scores	Criteria
1	Periapical structures appear normal with no pathological changes.
2	Minor alterations detected in the bone structure.
3	Noticeable bone structure changes accompanied by slight mineral loss.
4	Periapical periodontitis presenting with distinct radiolucent areas.
5	Advanced periapical periodontitis showing severe features and worsening characteristics.

### Statistical analysis

The numerical variables were examined for normality by evaluating their distribution and applying standard normality tests, including the Kolmogorov–Smirnov and Shapiro–Wilk tests. Age and PPD measurements followed a normal (parametric) distribution, whereas the remaining variables displayed non-parametric behavior. Accordingly, the data were summarized using mean, standard deviation (SD), median, and range values.

For variables that followed a parametric distribution, Student’s t-test was applied to compare the average age between the two groups. Repeated-measures ANOVA was performed to evaluate differences in PPD readings both between the groups and across the various time points within each group. When the ANOVA indicated a significant effect, Bonferroni’s post-hoc test was used to conduct pairwise comparisons.

For non-parametric variables, comparisons between the two groups were performed using the Mann–Whitney U test. Changes within each group over time were evaluated with Friedman’s test, and when this test showed significance, Dunn’s post-hoc test was applied for pairwise comparisons across the different time points. Categorical outcomes were summarized as frequencies and percentages, and group comparisons were conducted using the Chi-square test or Fisher’s Exact test when appropriate.

Agreement between the first and second examiners (intra-observer reliability), as well as between the two independent observers (inter-observer reliability), was assessed using the intraclass correlation coefficient (ICC). The relationship between the plaque index and the gingival index was analyzed using Pearson’s correlation coefficient. A significance threshold of *P* ≤ 0.05 was adopted. All statistical procedures were conducted using statistical software (IBM SPSS Statistics for Windows, Version 23.0; IBM Corp.; Armonk, NY, USA).

## Results

The study was conducted in accordance with the preregistered protocol (NCT05250609), and no changes were made to the intervention procedures or primary outcome measures.

A modified intention-to-treat (mITT) approach was used. Follow-up was complete in the Endocrown Group, while one participant in the SSC Group missed the 6-month, 9-month, and 12-month visits and was analyzed using available data.

### Baseline characteristics

An evaluation of the baseline variables for both groups demonstrated no significant differences in mean age (*p* = 0.097) or in gender distribution (*p* = 0.682). These findings are presented in Table 3.

**Table 3:** Summary of baseline characteristics, including mean and standard deviation (SD), frequency counts (n), percentage values, and the outcomes of Student’s t-test and Chi-square test used to compare the two groups

	Endocrown Group (*n* = 12)	SSC Group (*n* = 12)	*P*-value
Age (Years)			0.097
Mean (SD)	11 (0.95)	11.58 (0.67)	
Gender [n (%)]			0.682
Male	5 (41.7%)	6 (50%)	
Female	7 (58.3%)	6 (50%)	

* Significant at *P* ≤ 0.05

### Clinical evaluation

#### Plaque Index for Crowned Tooth (PI Score)

##### Comparison between groups

Analysis of PI scores for teeth restored with endocrowns and SSCs showed no significant differences at baseline or throughout the follow-up period (*p* > 0.05 for all comparisons). Although slight to moderate effect sizes appeared at certain follow-up points, these variations remained statistically non-significant. These findings are summarized in Table 4.

**Table 4:** Summary of descriptive statistics and the results of the Mann–Whitney U test comparing PI scores between the two groups for the crowned teeth, along with Friedman’s test results assessing within-group changes

Time	Endocrown Group (*n* = 12)		SSC Group (*n* = 12)		*P*-value	Effect size (d)
	Median (Range)	Mean (SD)	Median (Range)	Mean (SD)		
Pre-operative	1.75 (0.75-3) ^A^	1.88 (0.69)	1.88 (1–3) ^A^	1.83 (0.62)	0.930	0.035
1 week	1.5 (0.25–2.5) ^B^	1.54 (0.72)	1.38 (0–3) ^B^	1.35 (0.86)	0.502	0.274
3 months	1.25 (0.5-2) ^B^	1.1 (0.51)	1 (0-1.5) ^B^	0.9 (0.62)	0.494	0.274
6 months	1 (0–3) ^B^	1.06 (0.87)	1.25 (0–3) ^B^	1.39 (0.8)	0.142	0.742
9 months	1.25 (0.5-3) ^B^	1.25 (0.71)	1 (0–2) ^B^	0.89 (0.66)	0.335	0.524
12 months	1.38 (0.5–2.5) ^B^	1.4 (0.58)	1 (0–2) ^B^	1.07 (0.71)	0.306	0.55
*P*-value	0.001*		0.020*			
Effect size (w)	0.338		0.223			

* Indicates significance at *P* ≤ 0.05

Variations in superscript letters within the same column denote statistically significant changes over time

##### Changes within each group

Both groups demonstrated a significant decline in PI scores throughout the study period (Endocrown Group: *p* = 0.001, effect size = 0.338; SSC Group: *p* = 0.020, effect size = 0.223). Pairwise comparisons showed that PI values dropped significantly after the first week in both groups. However, no significant differences were detected between the groups at later follow-up intervals, as summarized in Table 4.

#### Plaque Index For Individual (PI Score)

##### Comparison between groups

Across all assessment points—baseline, 1 week, 3 months, 9 months, and 12 months—there were no significant differences in individual PI scores between the endocrown and SSC Groups (p > 0.05 for every comparison). At the 6-month evaluation, however, the Endocrown Group showed notably lower PI values than the SSC Group (p = 0.042, effect size = 1.035). These outcomes are detailed in Table 5.

**Table 5:** Summary of descriptive statistics and the results of the Mann–Whitney U test comparing PI scores between the two groups for the individual, along with Friedman’s test results assessing within-group changes

Time	Endocrown Group (*n* = 12)		SSC Group (*n* = 12)		*P*-value	Effect size (d)
	Median (Range)	Mean (SD)	Median (Range)	Mean (SD)		
Pre-operative	1.1 (0.2–2.5)	1.19 (0.64)	1.15 (0.54–2.13) ^A^	1.31 (0.49)	0.470	0.298
1 week	1.04 (0.58–2.5)	1.15 (0.55)	1.17 (0.21–1.75) ^A^	1.08 (0.46)	0.795	0.106
3 months	0.92 (0-1.5)	0.89 (0.44)	0.73 (0.13–1.33) ^B^	0.72 (0.41)	0.284	0.447
6 months	0.65 (0.25–2.21)	0.82 (0.54)	1.17 (0.42–1.88) ^A^	1.16 (0.43)	0.042*	1.035
9 months	0.96 (0.33–1.88)	1 (0.5)	0.79 (0.083–1.13) ^B^	0.75 (0.32)	0.217	0.644
12 months	0.96 (0.38–1.92)	0.99 (0.46)	1.21 (0.5-2) ^A^	1.12 (0.45)	0.497	0.409
*P*-value	0.510		0.020*			
Effect size (w)	0.071		0.223			

* Indicates significance at *P* ≤ 0.05

Variations in superscript letters within the same column denote statistically significant changes over time

##### Changes within each group

The Endocrown Group did not demonstrate significant shifts in PI values across the evaluation period (*p* = 0.510, effect size = 0.071). In contrast, the SSC Group showed notable variations over time (*p* = 0.020, effect size = 0.223). Specifically, PI scores declined from week 1 to month 3, increased from month 3 to month 6, decreased again between months 6 and 9, and finally rose from month 9 to month 12. These trends are presented in Table 5.

#### Gingival Index For Crowned Tooth (GI Score)

##### Comparison between groups

Across baseline, 3 months, 9 months, and 12 months, no significant group differences in GI scores were detected between the endocrown and SSC restorations (*p* > 0.05 for all assessments). Nevertheless, at the 1-week and 6-month evaluations, the Endocrown Group demonstrated markedly lower GI values than the SSC Group (*p* = 0.031, effect size = 0.829; and *p* = 0.004, effect size = 1.527, respectively). These results are summarized in Table 6.

**Table 6:** Summary of descriptive statistics and the results of the Mann–Whitney U test comparing GI scores between the two groups for the crowned teeth, along with Friedman’s test results assessing within-group changes

Time	Endocrown Group (*n* = 12)		SSC Group (*n* = 12)		*P*-value	Effect size (d)
	Median (Range)	Mean (SD)	Median (Range)	Mean (SD)		
Pre-operative	2 (0.5–2.5) ^A^	1.88 (0.48)	2 (0–3)	1.92 (0.87)	0.540	0.225
1 week	2 (1–2) ^A^	1.77 (0.33)	2 (1–3)	2.17 (0.58)	0.031*	0.829
3 months	1.88 (0–2) ^A^	1.6 (0.6)	1.5 (1-2.5)	1.63 (0.48)	0.738	0.13
6 months	1.5 (0–2) ^B^	1.23 (0.56)	2 (1.25–2.75)	1.87 (0.39)	0.004*	1.527
9 months	1 (0.5-2) ^B^	1.25 (0.54)	2 (1–2)	1.66 (0.42)	0.061	0.906
12 months	1.5 (0.5–2.75) ^B^	1.54 (0.63)	2 (1–3)	2.07 (0.56)	0.059	0.969
P-value	0.014*		0.084			
Effect size (w)	0.238		0.162			

* Indicates significance at *P* ≤ 0.05

Variations in superscript letters within the same column denote statistically significant changes over time

##### Changes within each group

The Endocrown Group demonstrated statistically significant variations in GI scores across the study period (*p* = 0.014, effect size = 0.238), with a notable reduction occurring between the 3-month and 6-month assessments. No additional significant changes were detected at other intervals. In contrast, the SSC Group showed no significant shifts in GI values over time (*p* = 0.084, effect size = 0.162). These findings are presented in Table 6.

#### Gingival Index For Individual (GI Score)

##### Comparison between groups

Evaluation of individual GI readings showed that the endocrown and SSC Groups did not differ significantly at any assessment point, including baseline and the follow-ups at 1 week, 3 months, 6 months, 9 months, and 12 months. These comparative outcomes are presented in Table 7.

**Table 7:** Summary of descriptive statistics and the results of the Mann–Whitney U test comparing GI scores between the two groups for the individual, along with Friedman’s test results assessing within-group changes

Time	Endocrown Group (*n* = 12)		SSC Group (*n* = 12)		*P*-value	Effect size (d)
	Median (Range)	Mean (SD)	Median (Range)	Mean (SD)		
Preoperative	1.21 (0.17–2.04) ^A^	1.19 (0.5)	0.87 (0.25–1.75) ^B^	0.95 (0.5)	0.285	0.447
1 week	1.12 (0.71–1.96) ^A^	1.22 (0.35)	1.21 (0.58–1.92) ^A^	1.19 (0.39)	0.954	0.024
3 months	1.15 (0.67–1.54) ^A^	1.11 (0.29)	1.06 (0.33–1.54) ^B^	1 (0.33)	0.562	0.237
6 months	1.33 (0.67–1.71) ^A^	1.24 (0.3)	1.29 (0.67–1.83) ^A^	1.33 (0.39)	0.600	0.347
9 months	0.92 (0-1.75) ^B^	0.95 (0.47)	1.08 (0.67–1.21) ^B^	0.97 (0.2)	0.804	0.237
12 months	1.33 (1.08–1.75) ^A^	1.36 (0.21)	1.33 (0.67–2.04) ^A^	1.29 (0.35)	0.600	0.347
P-value	0.033*		0.003*			
Effect size (w)	0.202		0.300			

* Indicates significance at *P* ≤ 0.05

Variations in superscript letters within the same column denote statistically significant changes over time

##### Changes within each group

The Endocrown Group demonstrated a significant overall shift in GI values across the study period (*p* = 0.033, effect size = 0.202). GI levels declined noticeably between the 6-month and 9-month assessments, then rose again from 9 to 12 months. No other intervals showed significant changes. These results are reported in Table 7.

The SSC Group also displayed significant variation in GI measurements over time (*p* = 0.008, effect size = 0.300). GI scores increased after the first week, declined from week 1 to month 3, increased again between months 3 and 6, decreased from 6 to 9 months, and finally rose once more by the 12-month follow-up, as summarized in Table 7.

### Correlation between Plaque Index and Gingival Index

A strong positive correlation was observed between PI and GI for both individual teeth and crowned teeth in both the endocrown and SSC Groups (Table). This indicates that as plaque accumulation increases, gingival inflammation tends to increase as well. All correlation values reached statistical significance (*p* < 0.05), indicating a strong linkage between the two measures. These findings are detailed in Table 8.

**Table 8:** Correlation between plaque index and gingival index for individuals and selected tooth in the Endocrown Group and the SSC Group

		Pearson Correlation	P value
Endocrown Group	For Individual	0.436	0.0001*
	For crowned Tooth	0.398	0.001 *
SSC Group	For Individual	0.283	0.016*
	For crowned Tooth	0.429	0.0001*

*Significant correlation as *P* < 0.05

#### Probing Pocket Depth (PPD in mm)

##### Comparison between groups

At baseline, the two groups showed comparable PPD values, with no significant statistical differences (*p* = 0.753, effect size = 0.705). At the subsequent assessments—1, 3, 6, 9, and 12 months—the Endocrown Group consistently recorded lower PPD measurements than the SSC Group, and these contrasts were statistically significant at each of these time points (*p* < 0.001; effect sizes: 0.573, 0.631, 0.705, 0.664, and 0.505, respectively). These comparisons are summarized in Table 9.

**Table 9:** Descriptive statistics and results of repeated measures ANOVA test for comparison between PPD (mm) in the two groups and the changes within each group

Time	Endocrown Group (*n* = 12)		SSC Group (*n* = 12)		*P*-value	Effect size (Partial Eta Squared)
	Mean	SD	Mean	SD		
Pre-operative	2.1 ^A^	0.34	2.16 ^C^	0.48	0.753	0.005
1 week	2 ^A^	0.3	2.91 ^B^	0.5	< 0.001*	0.573
3 months	1.9 ^A^	0.48	3.27 ^A^	0.62	< 0.001*	0.631
6 months	1.69 ^A^	0.48	3 ^B^	0.4	< 0.001*	0.705
9 months	1.42 ^B^	0.4	2.93 ^B^	0.7	< 0.001*	0.664
12 months	1.73 ^A^	0.49	2.86 ^B^	0.67	< 0.001*	0.505
*P*-value	0.004*		< 0.001*			
Effect size (*Partial Eta Squared*)	0.611		0.788			

* Indicates significance at *P* ≤ 0.05

Variations in superscript letters within the same column denote statistically significant changes over time

##### Changes within each group

Within the Endocrown Group, PPD values varied significantly over the follow-up period (*p* = 0.004, effect size = 0.611). A marked reduction was noted at the 9-month interval, followed by an increase at the 12-month visit. No additional time points demonstrated significant within-group changes. These trends are presented in Table 9.

The SSC Group showed notable variability in PPD measurements throughout the follow-up period (*p* < 0.001, effect size = 0.788). PPD values rose at the 1-week and 3-month evaluations, declined at 6 months, and did not demonstrate significant change at either the 9- or 12-month visits. These patterns are detailed in Table 9.

### Radiographic evaluation

#### Periapical Index (PAI)

##### Comparison between groups

Across all assessment intervals—baseline, 1 week, 6 months, and 12 months—the PAI scores of the Endocrown and SSC Groups did not differ in a statistically significant manner (*p* = 0.133, effect size = 0.617; *p* = 0.515, effect size = 0.249; *p* = 0.261, effect size = 0.485; and *p* = 0.242, effect size = 0.447, respectively). These comparative findings are presented in Table 10.

**Table 10:** Descriptive statistics and results of the Mann-Whitney U test for comparison between PAI scores in the two groups and Friedman’s test for the changes within each group

Time	Endocrown Group (*n* = 12)		SSC Group (*n* = 12)		*P*-value	Effect size (d)
	Median (Range)	Mean (SD)	Median (Range)	Mean (SD)		
Pre-operative	2.5 (2–5) ^A^	2.75 (0.97)	2 (1–5) ^A^	2.17 (1.34)	0.133	0.617
1 week	2 (1–3) ^A^	2 (0.74)	2 (1–5) ^A^	2 (1.28)	0.515	0.249
6 months	1 (1–2) ^B^	1.17 (0.39)	1 (1–4) ^B^	1.55 (0.93)	0.261	0.485
12 months	1 (1–2) ^B^	1.08 (0.29)	1 (1–2) ^B^	1.27 (0.47)	0.242	0.447
*P*-value	< 0.001*		0.008*			
Effect size (*w*)	0.783		0.357			

* Indicates significance at *P* ≤ 0.05

Variations in superscript letters within the same column denote statistically significant changes over time

##### Changes within each group

Both restorative groups demonstrated significant shifts in their PAI values (Endocrown: *p* < 0.001, effect size = 0.783; SSC Group: *p* = 0.008, effect size = 0.357). In each group, PAI scores dropped notably from the 1-week assessment to the 6-month follow-up, after which no further significant changes were detected at later time points. These trends are summarized in Table 10.

### Reliability evaluation of periapical index

Most intraobserver/interobserver reliability measurements show statistical significance (*p* < 0.05), indicating that the observed reliability is unlikely to be due to chance, as shown in Table 11.

**Table 11:** Intraobserver and interobserver reliability for the Endocrown Group and the SSC Group assessed across multiple time intervals

Reliability	periapical index	Interval	Intraclass Correlation	95% Confidence Interval		P value
				Lower Bound	Upper Bound	
Intraobserver	Endocrown Group	first week	0.916	0.736	0.975	0.000*
		2 weeks	0.956	0.848	0.987	0.000*
		6 months	0.842	0.452	0.955	0.002*
		12 months	1.000	1.000	1.000	----
	SSC Group	first week	1.000	1.000	1.000	----
		2 weeks	1.000	1.000	1.000	----
		6 months	0.924	0.737	0.978	0.000*
		12 months	1.000	1.000	1.000	-----
Interobserver	Endocrown Group	first week	0.813	0.349	0.946	0.005*
		2 weeks	0.869	0.545	0.962	0.001*
		6 months	0.808	0.332	0.945	0.005*
		12 months	1.000	1.000	1.000	----
	SSC Group	first week	1.000	1.000	1.000	-----
		2 weeks	0.924	0.737	0.978	0.000*
		6 months	0.571	-0.489	0.877	0.08
		12 months	1.000	1.000	1.000	-----

*Significant reliability as *P* < 0.05

**Fig. 2:**
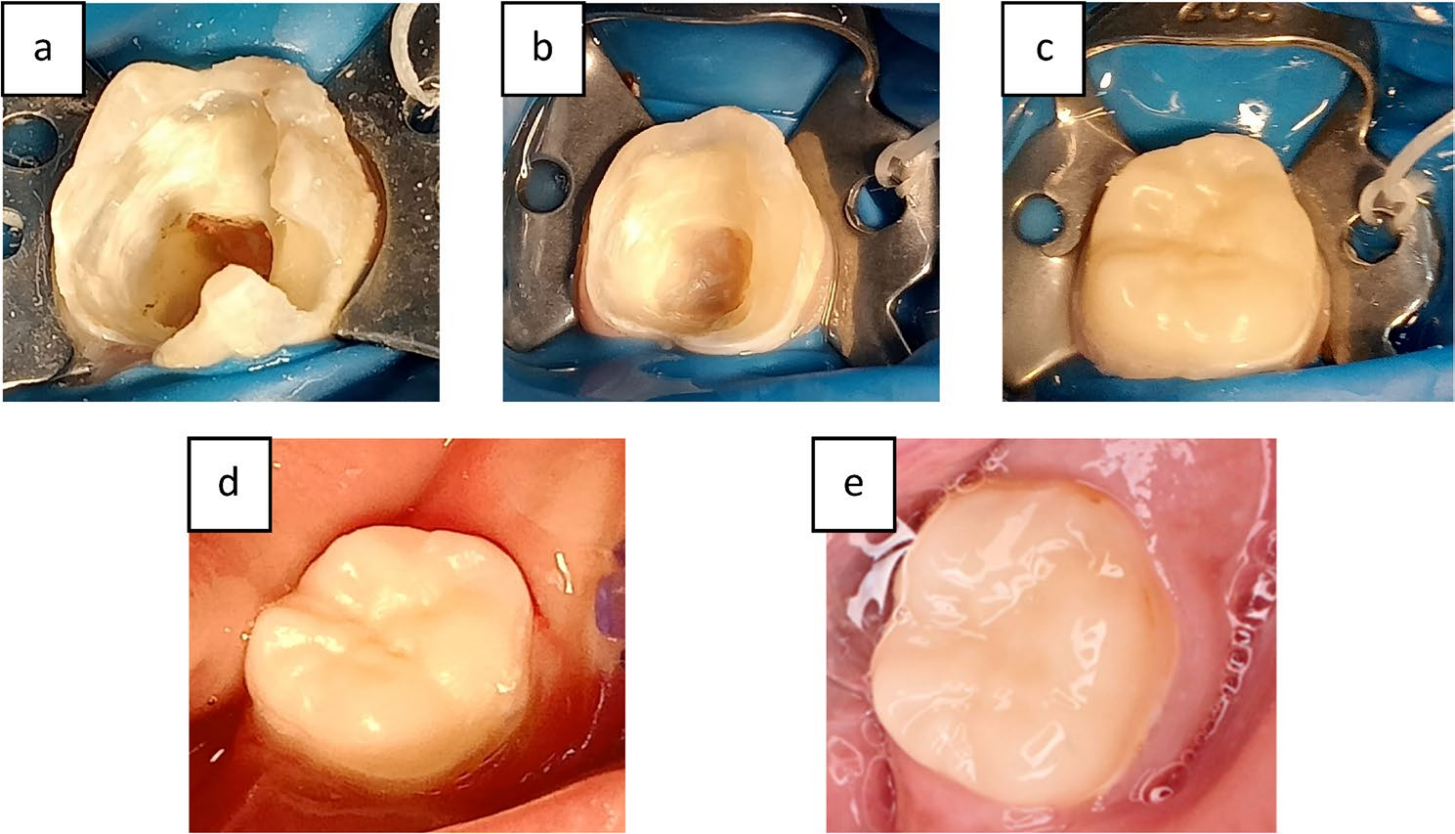
Clinical photographs of the Endocrown Group showing (**a**) preoperative, (**b**) after preparation, (**c**) after cementation of Endocrown, (**d**) immediate postoperative, (**e**) 12-month postoperative

**Fig. 3:**
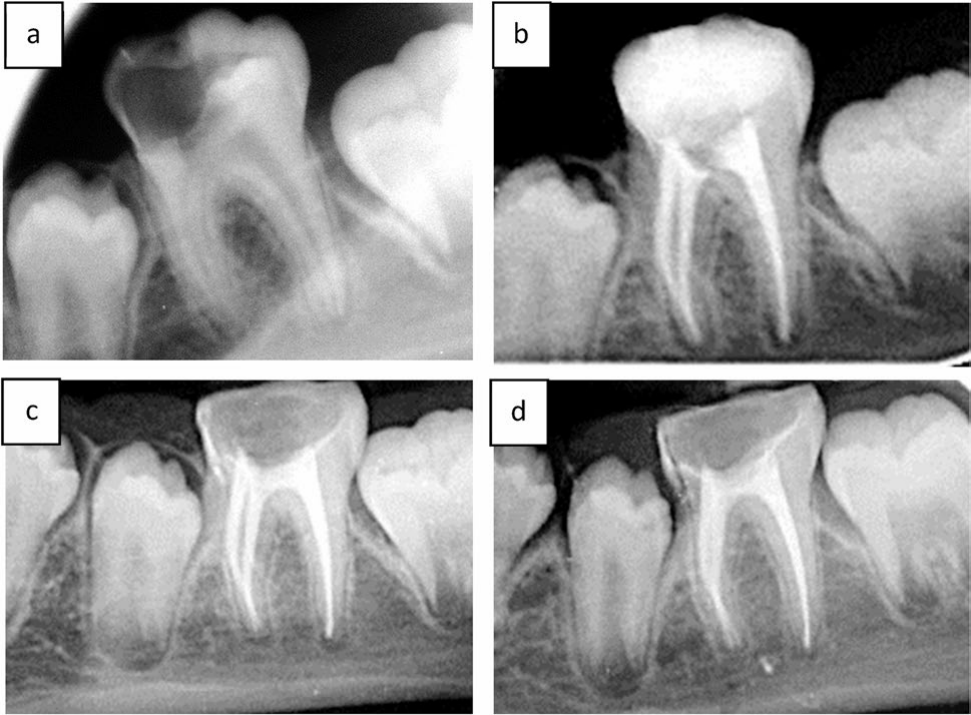
Radiographs of the Endocrown Group (**a**) preoperative, (**b**) after one week, (**c**) 6-month postoperative, (**d**) 12-month postoperative

**Fig. 4:**
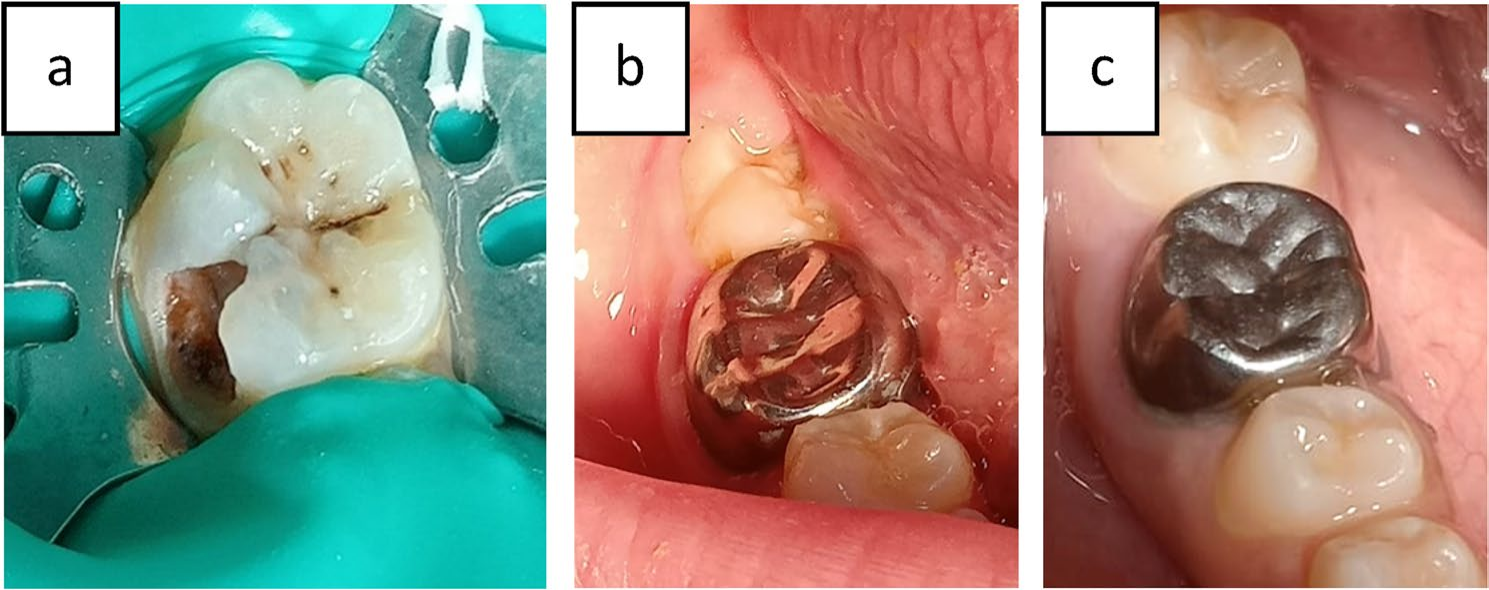
Clinical photos of the SSC Group (**a**) preoperative, (**b**) immediate postoperative, (**c**) 12-month postoperative

**Fig. 5:**
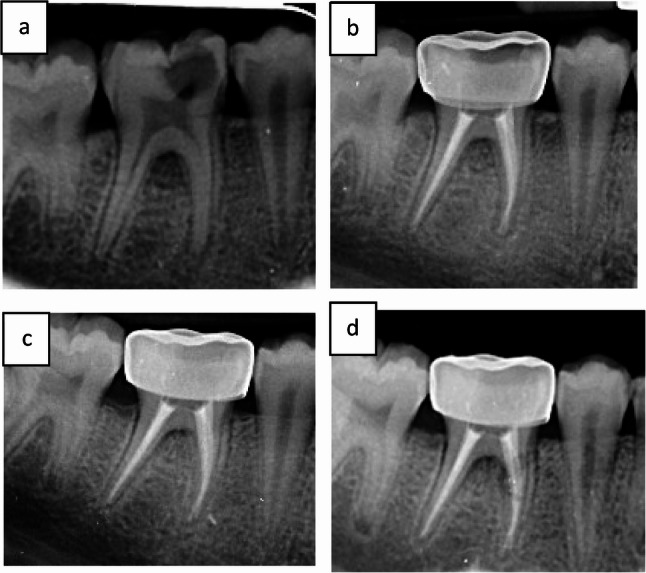
Radiographs of the SSC Group (**a**) preoperative, (**b**) after one week, (**c**) 6-month postoperative, (**d**) 12-month postoperative

Representative clinical photographs and radiographic images for both restorative approaches are presented in Figs. 2, 3, 4 and 5.

## Discussion

This randomized controlled trial primarily evaluated periodontal responses—particularly probing pocket depth (PPD)—and secondarily assessed plaque index (PI), gingival index (GI), and radiographic periapical status in endodontically treated first permanent molars restored with CAD/CAM endocrowns versus stainless steel crowns over a 12-month period. The results demonstrated significantly lower PPD values in the Endocrown Group throughout follow-up, with improvements in gingival parameters at selected time points, while plaque levels and radiographic healing were comparable between the two restorative approaches. These findings led to rejection of the primary null hypothesis and partial rejection of the secondary null hypothesis, as significant differences were observed in key periodontal parameters, particularly probing pocket depth.

The periodontal differences observed in this study may be related to the distinct preparation designs and restorative protocols used. Endocrowns were prepared with supragingival butt-joint margins and adhesively bonded, preserving cervical tooth structure and reducing periodontal irritation. In contrast, SSC required circumferential reduction with margins placed slightly subgingivally and were cemented with glass ionomer, which may favor plaque retention. Biomechanically, adhesive retention within the pulp chamber allows improved stress distribution and internal reinforcement of the remaining tooth structure [16]. Additionally, the composite CAD/CAM material exhibits favorable elastic properties that may reduce stress transmission to surrounding periodontal tissues (16). These combined factors likely contributed to the variations in periodontal response between groups.

This study indicated the impact of composite CAD/CAM Endocrowns versus SSCs on plaque accumulation and gingival health in early adolescents. The Endocrown Group displayed consistently low PI scores throughout the study period for both individual and crowned teeth. This suggests that Endocrowns may effectively reduce plaque accumulation around the restoration margins. In contrast, the SSC Group exhibited fluctuating PI scores for the individual, which may indicate potential challenges with plaque control around these restorations.

This study’s plaque index findings disagreed with those of Talekar et al. 2023, who reported significant plaque accumulation at one week in both PZCs and SSCs, followed by a gradual decrease. Talekar et al. attributed this to gingival irritation and trauma during crown preparation, which likely increased sensitivity during brushing, leading children to avoid cleaning the area [25]. In contrast, this study observed a significant decrease in plaque scores at one week in both composite CAD/CAM Endocrowns and SSCs, followed by no significant changes, likely due to the emphasis on oral hygiene instructions.

The results of this study are partially consistent with those of Geduk et al. 2023, who found that PI scores for SSCs remained stable, while PZCs consistently had lower PI scores than those for SSCs throughout the study period. Regarding GI, SSCs showed higher scores at follow-ups, with a significant decrease only at 18 months. In contrast, PZCs displayed significant GI reduction at 18 months. Furthermore, PZCs consistently exhibited lower GI scores than SSCs [10]. Similar to the present study, Geduk et al. reported no improvement in oral hygiene despite regular follow-ups and patient motivation.

The findings of this study are in agreement with Muñoz-Sánchez et al. 2021, who reported that over one-third of stainless-steel crowns placed on permanent molars exhibited marginal discrepancies—such as open margins or ledging—which are known to act as plaque-retentive sites and potentially contribute to gingival inflammation [8]. Placing the SSC margins subgingivally may also present a risk of breaching the biological width of the periodontal attachment.

The finding of this study is contradicted by Koleventi et al. 2018, who reported significant increases in the degree of GI for the SSC Group at one week and six months (median: 0.85, range: 0-1.25, median: 1.37, range: 0–2, respectively) [26]. While this study showed no statistical changes in the degree of GI for the SSC Group at similar time points (median: 2.0, range: 1–3 at one week, median: 2.0, range: 1.25–2.75 at six months). Similarly, PPD in the present study showed no statistical changes for the SSC Group at one week and six months (mean: 2.91 mm, SD = 0.5 mm; mean: 3.0 mm, SD = 0.4 mm, respectively), whereas Koleventi et al., observed significant increases in PPD for their SSC Group (median: 2.0 mm, range: 2-2.25 at one week, median 2.37 mm, range: 2.25-3 at six months) [26].

Notably, both this study and Koleventi et al., found no changes in PI scores for the SSC Group at one week and six months. However, inflammation and deep pocket depth are present in both studies, mainly due to a lack of compliance with oral hygiene practices, and the subgingival margin of SSCs contributes to the increase in pocket depth.

The results of this study demonstrated that composite CAD/CAM Endocrowns had significantly lower post-treatment PPD than SSCs, despite no pre-operative differences. This may be attributed to the supragingival placement of Endocrown margins, minimizing potential periodontal tissue violation and inflammation associated with subgingival SSC margins.

Conversely, the findings of this study disagreed with those findings by Heidari et al. 2019 who revealed that in 23 FPMS treated with SSCs, there was a significant decrease in PPD in the mesial regions following six months of SSC placement. However, there was no notable difference in PPD after six months of SSC placement in the distal regions. Heidari et al., attributed these findings to the smooth surface of SSCs, which prevents plaque accumulation, and the children’s ability to better clean the mesial surface [5].

Radiographic evaluation revealed the healing of periapical disease following root canal treatment within each group. The 1-week radiographic assessment primarily served to establish a postoperative reference point, as periapical healing is not radiographically detectable at such an early stage. However, one case exhibited periapical inflammation (PAI of 2) caused by a fractured and debonded composite CAD/CAM Endocrown. Successful retreatment was performed for this case. Additionally, one patient with an SSC reported pain at 3 months despite normal X-rays—suspected cement irritation. The SSC was removed, and retreatment with a new crown was offered to this patient, but the parent declined further treatment.

The findings of this trial were consistent with Sigal et al. 2020 who observed positive radiographic outcomes, including bone health and periapical healing, in a special health care needs population treated with SSCs [27]. However, this finding partially agrees with Alassar et al. 2022 who reported successful healing in all cases. However, their direct composite group had only five cases, with two showing a PAI of 2, possibly due to bulk-fill composite shrinkage. In contrast, all IPS e.max CAD Endocrown cases exhibited complete healing (PAI 1), likely attributed to accurate marginal seal, no ceramic shrinkage, and better fracture resistance [18].

A prospective study was conducted by Geduk et al. 2023 who performed radiological assessments of the marginal adaptation of crowns and periapical pathology of teeth restored with PZCs and SSCs over an 18-month follow-up period. Similar to this study, their findings showed no periapical pathology. However, in contrast to our findings, they did observe slight overhangs or undercuts of the crown (< 1 mm ledging) in four PZCs and one SSC out of 48 crowns [10].

Conversely, two retrospective studies investigated the long-term outcomes of SSCs. Discepolo and Sultan. 2017 who reported an 11.6% failure rate (18/155) over 6–99 months. Failures included periodontal defects, impaction of adjacent teeth, defective crowns, debonding, and periapical pathology [28]. Similarly, Oh et al. 2020 who found a 15.7% failure rate (18/115) over 12–180 months, attributed it to defective restorations, debonding, periapical disease, and wear-related perforations [29].

To sum up, this study found that CAD/CAM Endocrowns represent a more favourable interim restoration for treated first permanent molars in pediatric patients compared with traditional SCCs. Their supragingival margins appear to contribute to better periodontal health, supporting their use as a biologically favourable restorative choice in pediatric dentistry.

Although the trial was conducted within a university hospital, the Pediatric Dentistry Department at Cairo University is the largest national centre and receives patients from all regions of Egypt, which supports the broader applicability of the findings. Nonetheless, the study population consisted of cooperative children aged 10–13 years and all procedures were performed by a single operator, which may not represent every clinical scenario. Even so, the favourable periodontal response associated with the supragingival margins of CAD/CAM Endocrowns suggests that comparable outcomes may be expected in similar pediatric settings.

One notable constraint of the present investigation is the brief duration of follow-up, which limited the ability to observe how the restorations perform over longer periods. Studies designed with extended monitoring intervals would help clarify long-term outcomes. In addition, the economic implications of using CAD/CAM Endocrowns versus stainless-steel crowns were not analyzed, and future work should address this aspect to better inform clinical choices.

## Conclusion

Over the 12-month observation period, composite CAD/CAM endocrowns were associated with more favorable periodontal parameters than stainless-steel crowns in endodontically treated first permanent molars. Both restorations showed satisfactory radiographic healing. Composite CAD/CAM endocrowns may be considered a viable restorative alternative for pediatric patients requiring post-endodontic rehabilitation of permanent molars.

### Recommendation

- Examining the financial implications of using CAD/CAM Composite Endocrowns relative to conventional restorative options is needed, as this information would assist clinicians and healthcare providers in making more informed treatment choices.
- Additional long-term, follow-up investigations are essential to verify the durability, clinical behavior, and periodontal impact of CAD/CAM Composite Endocrowns in children.

## Supplementary Information

Supplementary Material 1.

Supplementary Material 2.

Supplementary Material 3.

Supplementary Material 4.

Supplementary Material 5.

## Data Availability

All data relevant to this study are presented in the main article and its supplementary materials. For further inquiries or to request additional details, the corresponding author, Dr. Basheer Ali (Basheer.ali@dentistry.cu.edu.eg), may be contacted.

## Abbreviations

FPMs: First Permanent Molars
SSCs: Stainless Steel Crowns
PZCs: Preformed Zirconia Crowns
PI: Plaque Index
PPD: Probing Pocket Depth
PAI: Periapical Index
RCT: Root Canal Treatment
CAD/CAM: Computer-Aided Design/Computer-Aided Manufacturing
mITT: modified intention-to-treat
ICC: Intraclass Correlation Coefficient
VPS: Vinyl Polysiloxane
LED: Light-Emitting Diode
