# Supplementary material for: Periodontal health of endodontically treated molars restored with composite CAD/CAM endocrowns versus stainless steel crowns in Egyptian children: a randomized controlled trial
Source: BMC Oral Health. 2026 Apr 18;26:985. doi: 10.1186/s12903-026-08077-0 (PMC13248274; doi:10.1186/s12903-026-08077-0)
Supplement: Supplementary file 1 — Supplementary Material 1. [file 12903_2026_8077_MOESM1_ESM.pdf]

Cairo University
Faculty of Dentistry
Research Ethics Committee

جامعه القاهرة
كلية طب الأسنان
لجنه أخلاقيات البحث العلمي

# To whom it may concern,

The following research protocol has been approved by the Research Ethics Committee, Faculty of Dentistry, Cairo University on 29th of March 2022 with approval number 4-3-22.

With the following research title: -

Clinical and radiographic evaluation of resin endocrown as long-term interim restoration versus stainless steel crown for endodontically treated first permanent molars in children: Randomized controlled pilot study.

The name of the principal investigator was Basheer Ali Mabkhot.

We declare that our committee for Human Subject Research at "Faculty of Dentistry, Cairo University" is organized and operated according to the Declaration of Helsinki for human subject research (2013).

Head of the committee

Date 21 /1 /2025

Prof. Olfat Hassanein

11 Elsaraya Street , Manial , Cairo, Egypt
Tel.(002) (02) 23642705/ 23642483/ Ext 543
Fax(002) (02) 23646375

١١شارع السرايا – المنيل - القاهرة
ت ٢٣٦٤٢٧٠٥ - ٢٣٦٤٢٤٨٣ - داخلى (٥٤٣)
فاكس ٢٣٦٤٦٣٧٥

Email: rec@dentistry.cu.edu.eg
