# Supplementary material for: Periodontal health of endodontically treated molars restored with composite CAD/CAM endocrowns versus stainless steel crowns in Egyptian children: a randomized controlled trial
Source: BMC Oral Health. 2026 Apr 18;26:985. doi: 10.1186/s12903-026-08077-0 (PMC13248274; doi:10.1186/s12903-026-08077-0)
Supplement: Supplementary file 3 — Supplementary Material 3. [file 12903_2026_8077_MOESM3_ESM.docx]

Sample size calculation

**Probing Pocket Depth:**

Sample size calculated depending on this pilot study as reference. According to this study, the minimally accepted sample size was 6 per group, when mean ± standard deviation of pocket depth after 12 months in group A was 1.73 ± 0.49 while in group B mean ± standard deviation was 2.86 ± 0.67, with 1.92 effect size when the power was 80 % & type I error probability was 0.05. Total sample size increased to 8 per group to compensate for the 20% drop out. The t test was performed by using G.power3.1.9.7.

***Power curve***
