# Supplementary material for: Periodontal health of endodontically treated molars restored with composite CAD/CAM endocrowns versus stainless steel crowns in Egyptian children: a randomized controlled trial
Source: BMC Oral Health. 2026 Apr 18;26:985. doi: 10.1186/s12903-026-08077-0 (PMC13248274; doi:10.1186/s12903-026-08077-0)
Supplement: Supplementary file 4 — Supplementary Material 4. [file 12903_2026_8077_MOESM4_ESM.docx]

1. Statistical analysis

Statistical analysis was performed with SPSS 20®, Graph Pad Prism® and Microsoft Excel 2016. Reliability between 1^st^ read and 2^nd^ read (intraobserver reliability) and between 1^st^ observer and 2^nd^ observer (Interobserver reliability) was permed by using Kappa test (ICC). Correlation between plaque index and gingival index was performed by using Pearsons Correlation coefficient. All data were presented in ( ) tables & ( ) graphs.

1. Reliability evaluation of periapical index (table 1):

**Intraobserver Reliability:**

- Group A shows high intraobserver reliability across all time points, with ICC values ranging from 0.842 to 1.000.
- Group B demonstrates perfect intraobserver reliability (ICC = 1.000) for all time points except at 6 months (ICC = 0.924).
- Both groups show statistically significant reliability (p < 0.05) when not perfect (ICC ≠ 1.000).

**Interobserver Reliability:**

- Group A's interobserver reliability is generally lower than its intraobserver reliability, with ICC values ranging from 0.808 to 1.000.
- Group B shows perfect interobserver reliability (ICC = 1.000) in the first week and 12 months, but lower values at 2 weeks (0.924) and 6 months (0.571).
- Most interobserver measurements are statistically significant (p < 0.05), except for Group B at 6 months (p = 0.08).

**Group Comparison:**

- Group B generally shows higher reliability than Group A, with more instances of perfect reliability (ICC = 1.000). However, Group B also shows the lowest overall reliability score (ICC = 0.571) for interobserver reliability at 6 months.

**Statistical Significance:**

- Most measurements show statistical significance (p < 0.05), indicating that the observed reliability is unlikely to be due to chance.

**Table (1): Intraobserver and interobserver reliability in Group A and B at different intervals:**

| Reliability | periapical index | Interval | Intraclass Correlation | 95% Confidence Interval | | P value |
| --- | --- | --- | --- | --- | --- | --- |
|  |  |  |  | Lower Bound | Upper Bound |  |
| Intraobserver | Group A | first week | 0.916 | 0.736 | 0.975 | 0.000* |
|  |  | 2 weeks | 0.956 | 0.848 | 0.987 | 0.000* |
|  |  | 6 months | 0.842 | 0.452 | 0.955 | 0.002* |
|  |  | 12 months | 1.000 | 1.000 | 1.000 | ---- |
|  | Group B | first week | 1.000 | 1.000 | 1.000 | ---- |
|  |  | 2 weeks | 1.000 | 1.000 | 1.000 | ---- |
|  |  | 6 months | 0.924 | 0.737 | 0.978 | 0.000* |
|  |  | 12 months | 1.000 | 1.000 | 1.000 | ----- |
| Interobserver | Group A | first week | 0.813 | 0.349 | 0.946 | 0.005* |
|  |  | 2 weeks | 0.869 | 0.545 | 0.962 | 0.001* |
|  |  | 6 months | 0.808 | 0.332 | 0.945 | 0.005* |
|  |  | 12 months | 1.000 | 1.000 | 1.000 | ---- |
|  | Group B | first week | 1.000 | 1.000 | 1.000 | ----- |
|  |  | 2 weeks | 0.924 | 0.737 | 0.978 | 0.000* |
|  |  | 6 months | 0.571 | -0.489 | 0.877 | 0.08 |
|  |  | 12 months | 1.000 | 1.000 | 1.000 | ----- |

*Significant reliability as P<0.05.

1. Correlation between plaque index and gingival index:

Table 2 and figure 1 -4 present correlations between plaque index and gingival index in two groups (endocrowns and SSCs). Measurements are taken for both individuals and selected teeth. Pearson correlation coefficient is used to measure the strength of the relationship between plaque and gingival indices. All correlations are positive, indicating that as plaque index increases, gingival index tends to increase as well.

**Group A Results:**

- For individuals: Moderate correlation (r = 0.436, p = 0.0001)

- For selected teeth: Weak to moderate correlation (r = 0.398, p = 0.001)

- Both correlations are statistically significant (p < 0.05)

**Group B Results:**

- For individuals: Weak correlation (r = 0.283, p = 0.016)

- For selected teeth: Moderate correlation (r = 0.429, p = 0.0001)

- Both correlations are statistically significant (p < 0.05)

**Comparison Between Groups:**

- Group A shows stronger correlation for individuals compared to Group B.

- Group B shows stronger correlation for selected teeth compared to Group A.

**Individual vs. Selected Tooth:**

- In Group A, the correlation is stronger for individuals than for selected teeth.

- In Group B, the correlation is stronger for selected teeth than for individuals.

**Statistical Significance:**

- All correlations are statistically significant (p < 0.05), indicating that the observed relationships are unlikely to be due to chance.

**Table (2): Correlation between plaque index and gingival index for individuals and selected tooth in Group A and Group B:**

|  |  | Pearson Correlation | P value |
| --- | --- | --- | --- |
| Group A | For Individual | 0.436 | 0.0001* |
|  | For Selected Tooth | 0.398 | 0.001 * |
| Group B | For Individual | 0.283 | 0.016* |
|  | For Selected Tooth | 0.429 | 0.0001* |

**Significant correlation as P<0.05.*

Figure (1): Scattered chart representing correlation between plaque index and gingival index regaring individual in group A.

Figure (2): Scattered chart representing correlation between plaque index and gingival index regaring selected tooth in group A.

Figure (3): Scattered chart representing correlation between plaque index and gingival index regaring individual in group B.

Figure (4): Scattered chart representing correlation between plaque index and gingival index regaring selected tooth in group B.
